# Outcomes of patients aged 70 years or younger with aggressive ATL at core hospitals for ATL treatment in Tokyo

**DOI:** 10.1007/s12185-025-04057-2

**Published:** 2025-09-02

**Authors:** Junya Makiyama, Nobuhiro Ohno, Koji Jimbo, Toyotaka Kawamata, Kazuaki Yokoyama, Takaaki Konuma, Seiko Kato, Tomonari Takemura, Ayumu Ito, Takashi Tanaka, Yoshihiro Inamoto, Shigeo Fuji, Yoichi Imai, Satoshi Takahashi, Yasuhito Nannya, Arinobu Tojo, Takahiro Fukuda, Kaoru Uchimaru

**Affiliations:** 1 Department of Hematology/Oncology, Research Hospital, The Institute of Medical Science, The University of Tokyo, 4-6-1 Shirokanedai, Minato-ku, Tokyo, Japan; 2 Department of Hematology, Sasebo City General Hospital, Nagasaki, Japan; 3 Department of Hematology, Kanto Rosai Hospital, Kanagawa, Japan; 4 Department of Hematology, Tokyo Metropolitan Bokutoh Hospital, Tokyo, Japan; 5 Department of Hematopoietic Stem Cell Transplantation, National Cancer Center Hospital, Tokyo, Japan; 6 Department of Hematology, Osaka International Cancer Center, Osaka, Japan; 7 Department of Hematology and Oncology, Dokkyo Medical University, Tochigi, Japan; 8 Division of Molecular Therapy, Advanced Clinical Research Center, The Institute of Medical Science, The University of Tokyo, Tokyo, Japan; 9 Division of Clinical Precision Research Platform, The University of Tokyo, Tokyo, Japan; 10 Institute of Science Tokyo, Tokyo, Japan; 11 Laboratory of Tumor Cell Biology, Department of Computational Biology and Medical Sciences, Graduate School of Frontier Sciences, The University of Tokyo, Tokyo, Japan

**Keywords:** Adult T-cell leukemia-lymphoma, Allogeneic hematopoietic stem cell transplantation, Non-endemic area

## Abstract

Adult T-cell leukemia-lymphoma (ATL) is one of the most intractable peripheral T-cell neoplasms caused by human T-cell leukemia virus type I (HTLV-1) infection. Recently, the incidence of HTLV-1 infection and ATL has increased in non-endemic metropolitan areas in Japan. This retrospective study evaluated the clinical features and outcomes of patients with aggressive ATL aged 70 years or younger treated at a core hospital in Tokyo between 2004 and 2016. The median follow-up was 124.4 months for survivors. Among the 71 patients, 46 (64.8%) underwent allogeneic hematopoietic stem cell transplantation (allo-HSCT). The 3 year overall survival rate was 45.7% in allo-HSCT group versus 0% in non-allo-HSCT group. Patients who achieved complete/partial remission before allo-HSCT had a significantly better survival rate than those with stable/progressive disease (51.4% vs 27.3%). The 2 year cumulative incidence of relapse/progression and non-relapse mortality after allo-HSCT was 41.3% and 21.7%, respectively. In this study, a large percentage of patients underwent allo-HSCT and achieved favorable outcomes. As cases continue to rise in metropolitan areas, core hospitals will play a critical role in ATL treatment.

**Supplementary Information:**

The online version contains supplementary material available at 10.1007/s12185-025-04057-2.

## Introduction

Adult T-cell leukemia-lymphoma (ATL) is one of the most intractable peripheral T-cell neoplasms caused by human T-cell leukemia virus type I (HTLV-1) infection [1–4]. Approximately 10 million people worldwide are estimated to be infected with HTLV-1 [5] with the main endemic areas including Japan, the Caribbean islands, Central and South America, Central and South Africa, parts of the Middle East, Melanesia, and Aboriginal regions of Australia [5]. Japan has a high prevalence of HTLV-1 infection and ATL, which are concentrated in the southwest regions (Kyushu and Shikoku islands) [6–8]. However, in recent years, the incidence of HTLV-1 infection and ATL has increased in non-endemic metropolitan cities such as Tokyo and Osaka [7, 9, 10]. Thus, the treatment of ATL is a critical issue for endemic areas, as well as non-endemic metropolitan cities.

ATL is classified into four subtypes according to the Shimoyama criteria: smoldering, chronic, lymphoma, and acute [11]. The smoldering and chronic types without unfavorable prognostic factors—such as high lactate dehydrogenase, high blood urea nitrogen, and low albumin concentration—are considered indolent types. In contrast, acute, lymphoma, and chronic types with unfavorable prognostic factors are classified as aggressive types. Although zidovudine and interferon-α are used as standard therapies for indolent ATL in Western countries [12–14], in Japan, most indolent ATL patients receive no therapy except for those who have severe skin lesions. Intensive combination chemotherapy is administered to those with aggressive ATL; however, most aggressive ATLs are resistant to conventional chemotherapeutic agents. Several clinical trials of ATLs have been conducted with unsatisfactory results. The vincristine, cyclophosphamide, doxorubicin, and prednisone (VCAP)-doxorubicin, ranimustine, and prednisone (AMP)-vindesine, etoposide, carboplatin, and prednisone (VECP) regimen was reported to be more effective than biweekly cyclophosphamide, doxorubicin, vincristine, and prednisone (CHOP); however, the median survival time (MST) of patients treated with VCAP-AMP-VECP was only 13 months [15]. Several reports have reported the effectiveness of allogeneic hematopoietic stem cell transplantation (allo-HSCT) for the treatment of aggressive ATL [16]. These treatment approaches have been incorporated into the Japanese Society of Hematology practical guidelines for ATL [17].

However, most ATL outcome studies were based in endemic areas or were nationwide studies [18, 19], with few reports of outcomes from non-endemic metropolitan cities [20, 21]. Given the ongoing population migration from endemic regions such as Kyushu to metropolitan cities such as Tokyo, an increasing number of ATL cases are now being managed in non-endemic settings [7]. Therefore, region-specific data from institutions in non-endemic metropolitan areas are essential to complement existing knowledge and improve our clinical understanding of ATL management in these settings. Our group is situated in core hospitals for ATL treatment in Tokyo, a non-endemic metropolitan city, and we actively perform allo-HSCT for eligible patients with ATL who are 70 years or younger. Here, we report the clinical outcomes of these patients.

## Subjects and methods

### Subjects

We evaluated patients with aggressive ATL aged 70 years or younger who received chemotherapy at the Research Hospital of the Institute of Medical Science, The University of Tokyo (Tokyo, Japan) between April 2004 and December 2016. The clinical subtypes of ATL were classified according to the Shimoyama criteria [11]. A diagnosis of ATL was made based on clinical features, the presence of anti-HTLV-1 antibodies, histologically and/or cytologically proven mature T-cell malignancy, and the monoclonal integration of HTLV-1 proviral DNA into tumor cells in evaluable cases.

### Chemotherapy and allogeneic hematopoietic stem cell transplantation

Patients with aggressive ATL, if eligible, were treated with the VCAP-AMP-VECP regimen. Eligibility for this regimen was primarily determined by the attending physician based on various clinical factors. Patients who were not candidates for the VCAP-AMP-VECP regimen received a CHOP/CHOP-like regimen [22] or other treatments. For patients aged 70 years or younger who may be eligible for allo-HSCT, we initiated the search for a related donor after starting chemotherapy as soon as possible, or we coordinated immediately with the Japan Marrow Donor Program (JMDP) if the patients did not have a related donor. The response to induction chemotherapy and disease status at the time of allo-HSCT were classified as complete remission (CR), partial remission (PR), stable disease (SD), and progressive disease (PD) according to the criteria of the International Consensus Meeting published in 2009 [13]. Patients who had an appropriate donor, no severe organ dysfunction, no severe active infection, and no central nervous system (CNS) involvement following chemotherapy were considered eligible for allo-HSCT at the Department of Hematopoietic Stem Cell Transplantation, National Cancer Center Hospital (Tokyo, Japan) and the Department of Hematology/Oncology, Research Hospital, The Institute of Medical Science, The University of Tokyo. The decision to proceed with allo-HSCT was made by the attending physician after considering various clinical factors. In this study, the non-allo-HSCT group was defined as patients who did not undergo allo-HSCT during their clinical course, regardless of initial treatment intention. Clinical data were retrospectively collected from the medical records of each patient and updated in April 2023. This study was approved by the Ethical Committee and Institutional Review Board of The Institute of Medical Science, The University of Tokyo, and the National Cancer Center Hospital (Tokyo, Japan).

### Statistical analysis

Comparisons between groups were performed using Fisher’s exact test, as appropriate, for categorical variables, and the Mann–Whitney *U*-test for continuous variables. Overall survival (OS) was defined as the time from the initial treatment or transplantation until death by any cause. Patients who remained alive at the time of the last follow-up visit were censored. OS probability was estimated using the Kaplan–Meier method, and groups were compared using the log-rank test. Relapse/progression was defined as the time from allo-HSCT to relapse or progression, censoring the last known survival date. Non-relapse mortality (NRM) was defined as the time from allo-HSCT until death without relapse or progression. The probabilities of relapse/progression and NRM were estimated using the cumulative incidence function, and differences between groups were estimated using the Gray test. In the competing risk model for NRM, relapse/progression was defined as a competing risk.

All statistical analyses were performed using EZR (Saitama Medical Center, Jichi Medical University, Saitama, Japan), a graphical user interface for R (version 4.1.0; The R Foundation for Statistical Computing, Vienna, Austria). EZR is a modified version of R commander (version 1.54), which was designed to add frequently used statistical functions to biostatistics [23]. All P-values were two-sided. Statistical significance was set at P < 0.05.

## Results

### Patient characteristics

**Fig. 1:**
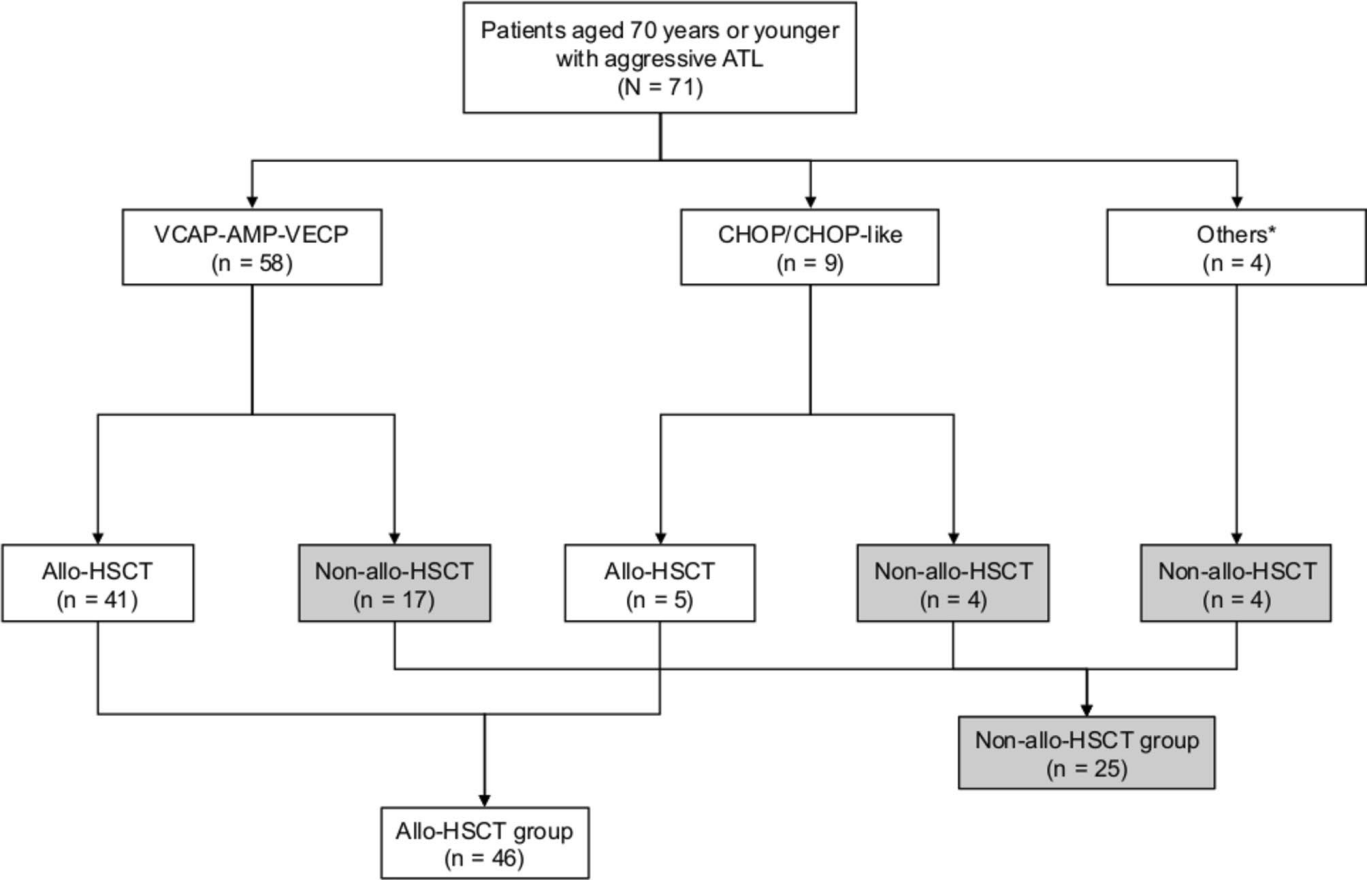
Flowchart of patients. Others*: Prednisone, dexamethasone, radiotherapy, or intrathecal administration of cytarabine, methotrexate, and prednisone. *allo-HSCT* allogeneic hematopoietic stem cell transplantation; *AMP* doxorubicin, ranimustine, and prednisone; *ATL* adult T-cell leukemia-lymphoma; *CHOP* cyclophosphamide, doxorubicin, vincristine, and prednisone; *VCAP* vincristine, cyclophosphamide, doxorubicin, and prednisone; *VECP* vindesine, etoposide, carboplatin, and prednisone

We conducted the study with 71 patients with aggressive ATL aged 70 years or younger. Forty-six (64.8%) patients underwent allo-HSCT (allo-HSCT group), and 25 (35.2%) did not (non-allo-HSCT group) (Fig. 1). The clinical characteristics of the patients in each group at the initial treatment are shown in Table 1. The median age was 59 years (range, 28–70 years) in the allo-HSCT group and 62 years (range, 38–70 years) in the non-allo-HSCT group. Forty-one patients (89.1%) in the allo-HSCT group and 20 patients (80.0%) in the non-allo-HSCT group were diagnosed with acute type disease. The Eastern Cooperative Oncology Group Performance Status (ECOG PS) score at initial treatment was significantly better in the allo-HSCT group than in the non-allo-HSCT group. Forty-one (89.1%) patients in the allo-HSCT group, and 17 (68.0%) patients in the non-allo-HSCT group, received the VCAP-AMP-VECP regimen. The modified ATL prognostic index (ATL-PI) [24] was evaluated in 54 patients but could not be assessed in the other 17 patients because of missing data for corrected serum calcium (n = 5), C-reactive protein (n = 3), and soluble interleukin-2 receptor (n = 9). Most patients were classified as having intermediate-risk according to the modified ATL-PI in both groups [30 (65.2%) patients in the allo-HSCT group and 13 (52.0%) patients in the non-allo-HSCT group]. There was no significant difference in the modified ATL-PI scores between the two groups.

**Table 1:** Patient characteristics at the initial treatment

Variable	Allo-HSCT (n = 46)	Non-allo-HSCT (n = 25)	*P*-value
Age (years), median (range)	59 (28–70)	62 (38–70)	0.12
≤ 55	13 (28.3)	5 (20.0)	0.57
> 55	33 (71.7)	20 (80.0)	
Sex			
Female	20 (43.5)	9 (36.0)	0.62
Male	26 (56.5)	16 (64.0)	
Subtype			
Acute	41 (89.1)	20 (80.0)	0.31
Lymphoma	2 (4.3)	4 (16.0)	
Chronic	3 (6.5)	1 (4.0)	
ECOG PS			
0–1	45 (97.8)	14 (56.0)	< 0.001
2–4	1 (2.2)	11 (44.0)	
Initial treatment			
VCAP-AMP-VECP	41 (89.1)	17 (68.0)	0.03
CHOP/CHOP like	5 (10.9)	4 (16.0)	
Others*	0	4 (16.0)	
Modified ATL prognostic index			
Low	5 (10.9)	1 (4.0)	0.30
Intermediate	30 (65.2)	13 (52.0)	
High	2 (4.3)	3 (12.0)	
Not evaluated	9 (19.6)	8 (32.0)	

Others*: Prednisone, dexamethasone, radiotherapy, or intrathecal administration of cytarabine, methotrexate, and prednisone

*allo-HSCT* allogeneic hematopoietic stem cell transplantation; *AMP* doxorubicin, ranimustine, and prednisone; *ATL* adult T-cell leukemia-lymphoma; *CHOP* cyclophosphamide, doxorubicin, vincristine, and prednisone; *ECOG PS* Eastern Cooperative Oncology Group Performance Status; *VCAP* vincristine, cyclophosphamide, doxorubicin, and prednisone; *VECP* vindesine, etoposide, carboplatin, and prednisone

Among the 25 patients in the non-allo-HSCT group, 22 were initially considered candidates for allo-HSCT and entered the coordination process but ultimately could not proceed. The main reasons for not receiving allo-HSCT were PD in 15 (60%) patients, CNS invasion in four (16%), infection in three (12%), comorbidity in two (8%), and no appropriate donor for one (4%). Among the 15 patients with PD, two patients were refractory to initial treatment, two patients experienced relapse after an initial response, and the remaining 11 patients had recurrent disease during the coordination period. Some patients had more than one reason for not receiving allo-HSCT. The remaining three patients opted not to undergo allo-HSCT.

Data of patients in the allo-HSCT group are summarized in Table 2. Forty-five (97.8%) patients had ECOG PS scores of 0–1 at allo-HSCT. Ten (21.7%) patients, primarily with residual or progressive disease, received mogamulizumab (Moga) treatment before allo-HSCT based on the decision of the attending physician. The median interval from the last Moga treatment to allo-HSCT was 69.5 days (range, 45–198 days). The disease status at allo-HSCT was CR/PR for 35 (76.1%) patients (10 CR patients and 25 PR patients) and SD/PD for 11 (23.9%) patients (7 SD patients and 4 PD patients). Thirty-one (67.4%) patients underwent allogeneic bone marrow transplantation from unrelated donors. The median interval from initial treatment to allo-HSCT was 175 days (range, 58–619 days). Regarding the preconditioning regimens for allo-HSCT, 5 (13.2%) patients received myeloablative conditioning, and 41 (86.8%) patients received reduced-intensity conditioning. Thirty patients (65.2%) received tacrolimus and short-term methotrexate for graft-versus-host disease. Engraftment was achieved in all patients.

**Table 2:** Characteristics of patients who underwent allo-HSCT

Variable	Number (%)/value
ECOG PS at the transplantation	
0–1	45 (97.8)
2–4	1 (2.2)
Pre-transplantation mogamulizumab	
Yes	10 (21.7)
Disease status at the transplantation	
CR/PR	35 (76.1)
SD/PD	11 (23.9)
Source of stem cells	
rBM	2 (4.3)
rPBSC	6 (13.0)
uBM	31 (67.4)
uPBSC	1 (2.2)
CB	4 (8.7)
Haplo-PBSC	2 (4.3)
Interval from initial treatment to allo-HSCT (days), median (range)	175 (58–619)
< 100	2 (4.3)
≥ 100	44 (95.7)
Conditioning regimen	
MAC	5 (13.2)
RIC	41 (86.8)
Prophylaxis of GVHD	
CSP	5 (10.9)
CSP/MTX	1 (2.2)
CSP/MMF	1 (2.2)
TAC/MTX	30 (65.2)
TAC/MMF	4 (8.7)
TAC/MTX/ATG	3 (6.5)
TAC/MTX/PTCy	1 (2.2)
TAC/MMF/PTCy	1 (2.2)

*allo-HSCT* allogeneic hematopoietic stem cell transplantation; *ATG* anti-thymocyte globulin; *CB* cord blood; *CR* complete remission; *CSP* cyclosporin; *ECOG PS* Eastern Cooperative Oncology Group Performance Status; *GVHD* graft versus host disease; *Haplo-PBSC* haploidentical peripheral blood stem cell; *MAC* myeloablative conditioning; *MMF* mycophenolate mofetil; *MTX* methotrexate; *PD* progressive disease; *PR* partial remission; *PTCy* posttransplant cyclophosphamide; *rBM* related bone marrow; *RIC* reduced-intensity conditioning; *rPBSC* related peripheral blood stem cell; *SD* stable disease; *TAC* tacrolimus; *uBM* unrelated bone marrow; *uPBSC* unrelated peripheral blood stem cell

### Survival analysis

**Fig. 2:**
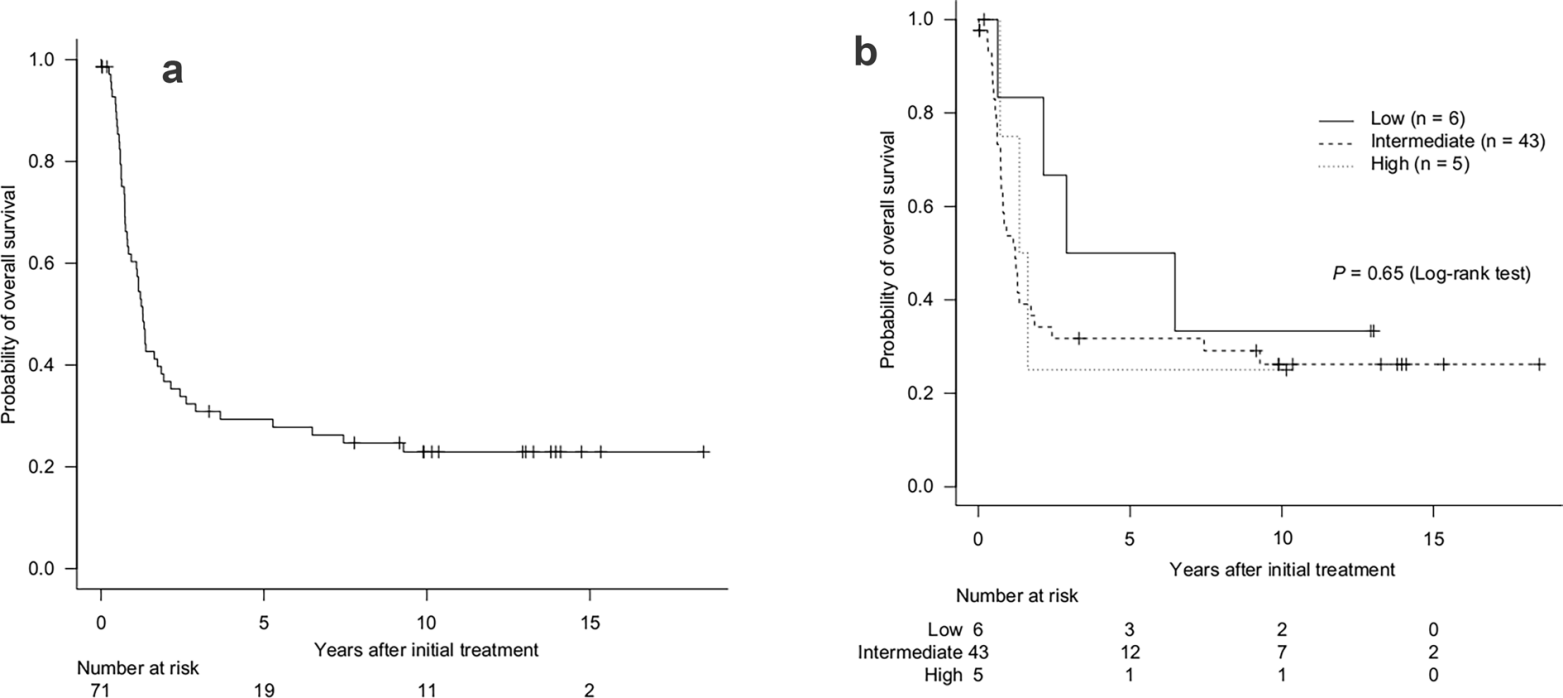
Survival of patients with ATL after initial treatment: entire cohort (**a**) and by modified ATL prognostic index (**b**). *ATL* adult T-cell leukemia-lymphoma

The median follow-up time for the survivors was 124.4 months (range, 0.4–222.0 months). For all 71 patients, the 3 year OS rate and the MST after initial treatment were 30.9% (95% confidence interval [CI] 20.4–42.0%) and 15.4 months (95% CI 10.2–22.2 months), respectively (Fig. 2a). Among the patients who survived for more than 5 years after initial treatment, four eventually died: three from ATL and one from a secondary malignancy. The 3 year OS rates and MST after initial treatment stratified by modified ATL-PI were as follows: 50.0% (95% CI 11.1–80.4%) and 56.3 months (95% CI 7.7 months–not evaluated) for patients with low-risk (n = 6); 31.7% (95% CI 18.3–46.0%) and 14.5 months (95% CI 8.9–22.2 months) for patients with intermediate-risk (n = 43); and 25.0% (95% CI 0.9–66.5%) and 17.9 months (95% CI 8.6 months–not evaluated) for patients with high-risk (n = 5) (Fig. 2b).

**Fig. 3:**
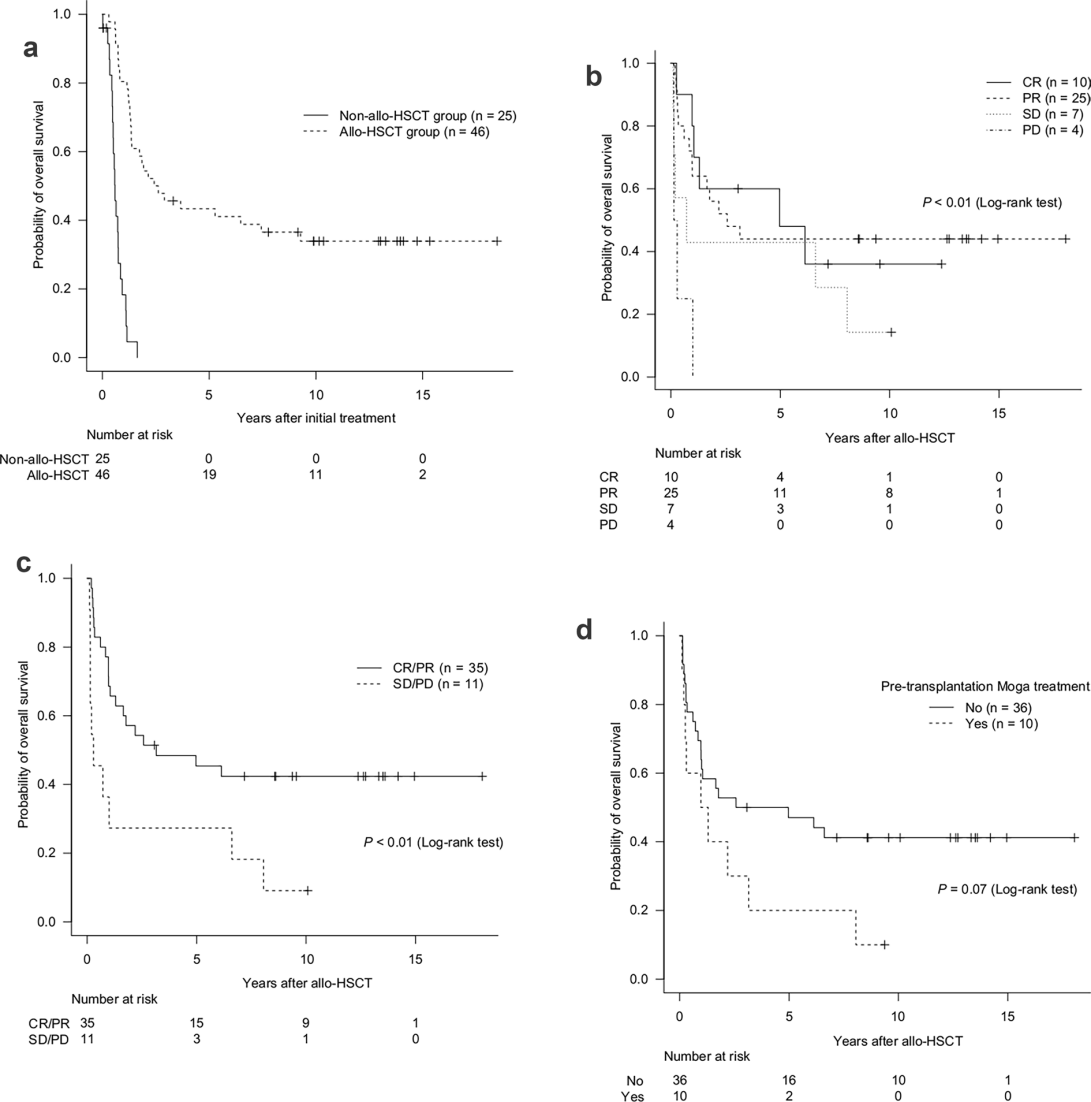
Survival of patients with aggressive ATL: by allo-HSCT (**a**), disease status at transplantation (**b**, **c**), and pre-transplantation Moga treatment (**d**). *allo-HSCT* allogeneic hematopoietic stem cell transplantation; *ATL* adult T-cell leukemia-lymphoma; *CR* complete remission; *Moga* mogamulizumab; *PD* progressive disease; *PR* partial remission; *SD* stable disease

In the allo-HSCT group, the 3 year OS rate after initial treatment was 45.7% (95% CI 31.0–59.2%). However, in the non-allo-HSCT group, the 3 year OS rate after the initial treatment was 0% (Fig. 3a). According to the disease status at allo-HSCT, the 3 year OS rate after allo-HSCT was 60.0% (95% CI 25.3–82.7%) in the CR group (n = 10), 48.0% (95% CI 27.8–65.6%) in the PR group (n = 25), 42.9% (95% CI 9.8–73.4%) in the SD group (n = 7), and 0% in the PD group (n = 4) (*P* < 0.01, log-rank test) (Fig. 3b). Patients with CR/PR had significantly better survival compared with those with SD/PD, with 3 year OS rates of 51.4% (95% CI 34.0–66.4%) vs 27.3% (95% CI 6.5–53.9%) (*P* < 0.01, log-rank test) (Fig. 3c). The 3 year OS rate after allo-HSCT was 50.0% (95% CI 32.9–64.9%) in patients who did not receive Moga treatment before allo-HSCT (n = 36) and 30.0% (95% CI 7.1–57.8%) in patients who received Moga treatment before allo-HSCT (n = 10) (*P* = 0.07, log-rank test) (Fig. 3d). Although not statistically significant, a trend towards a better 3 year OS was observed in patients who did not receive Moga treatment before allo-HSCT.

Additionally, we explored whether transplant outcomes changed over time. When patients were divided into two groups based on the transplant period (2005–2011 vs. 2012–2017), a trend toward worse OS was observed in the later cohort (*P* = 0.08, log-rank test) (Supplemental Fig. 1). Notably, in the later cohort, approximately one-third of patients received Moga before allo-HSCT.

**Fig. 4:**
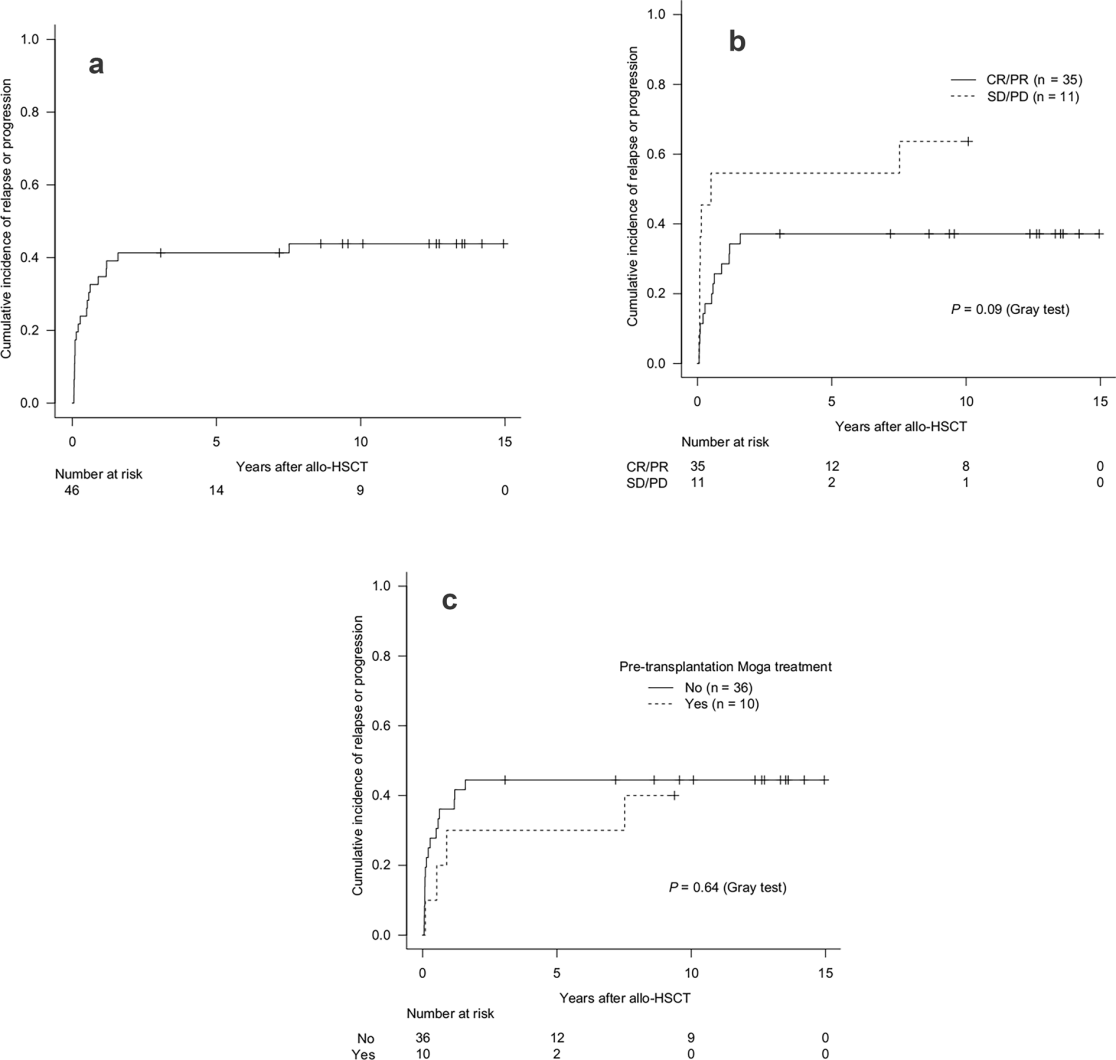
Cumulative incidence of relapse/progression: overall (**a**), by disease status at transplantation (**b**), and by pre-transplantation Moga treatment (**c**). *allo-HSCT* allogeneic hematopoietic stem cell transplantation; *CR* complete remission; *Moga* mogamulizumab; *PD* progressive disease; *PR* partial remission; *SD* stable disease

**Fig. 5:**
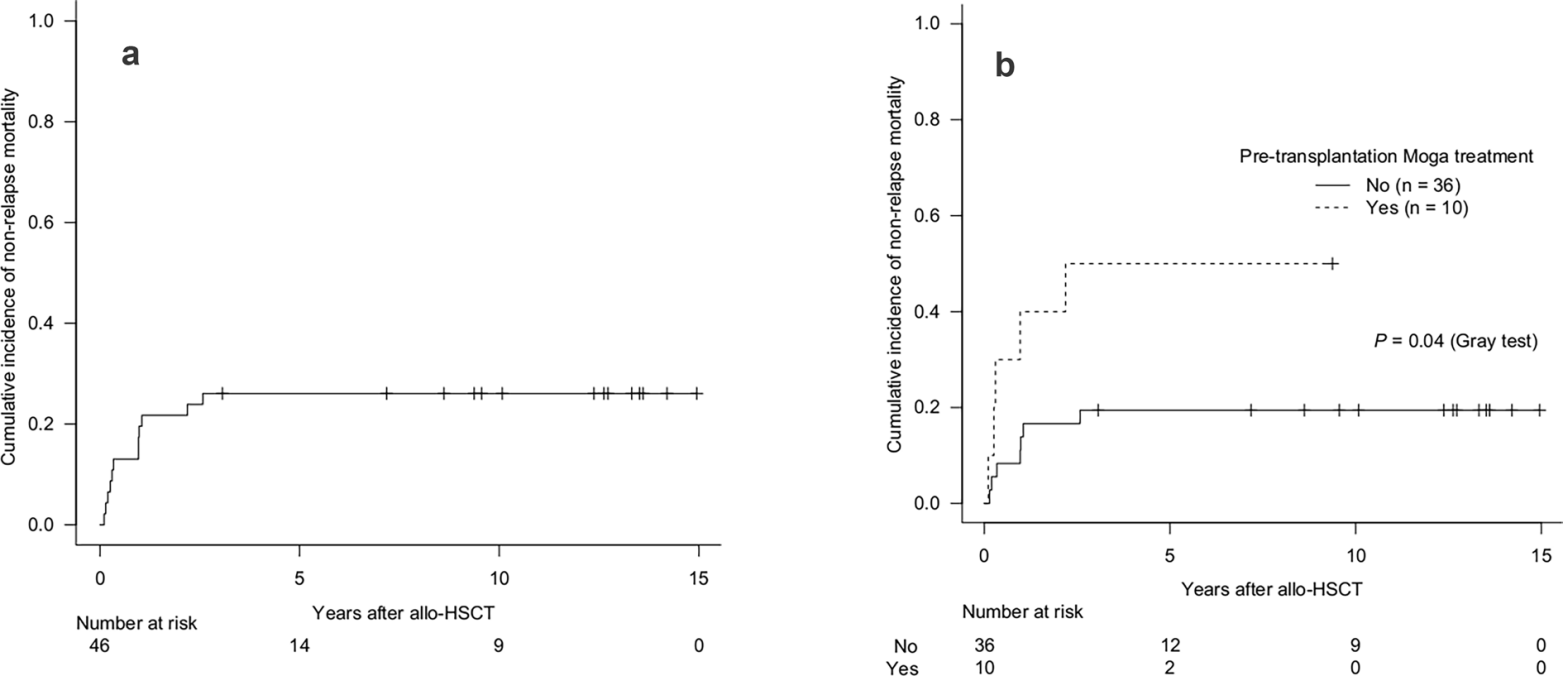
Cumulative incidence of non-relapse mortality: overall (**a**) and by pre-transplantation Moga treatment (**b**). *allo-HSCT* allogeneic hematopoietic stem cell transplantation; *CR* complete remission; *Moga* mogamulizumab; *PD* progressive disease; *PR* partial remission; *SD* stable disease

In the allo-HSCT group, the 2 year cumulative incidence of relapse/progression was 41.3% (95% CI 26.9–55.2%) (Fig. 4a). Patients with CR/PR at allo-HSCT had a 2 year cumulative incidence of relapse/progression of 37.1% (95% CI 21.3–53.0%), whereas those with SD/PD had a higher incidence of 54.5% (95% CI 19.5–79.9%) (*P* = 0.09, Gray test) (Fig. 4b). For patients who received pre-transplantation Moga treatment, the 2 year cumulative incidence of relapse/progression was 30.0% (95% CI 5.8–60.0%) compared with 44.4% (95% CI 27.6–60.0%) in those who did not (*P* = 0.64, Gray test) (Fig. 4c). The 2 year cumulative incidence of NRM was 21.7% (95% CI 11.1–34.7%) (Fig. 5a). Patients who received Moga treatment before allo-HSCT had a significantly higher 2 year cumulative incidence of NRM (40.0% [95% CI 10.3–69.2%]) compared with those who did not (16.7% [95% CI 6.6–30.7%]) (*P* = 0.04, Gray test) (Fig. 5b).

## Discussion

This retrospective study highlighted three important aspects related to the clinical features and outcomes of patients with ATL aged 70 years or younger in a non-endemic metropolitan city.

First, we achieved a high allo-HSCT implementation rate. Allo-HSCT was performed in 46 of 71 (64.8%) patients with aggressive ATL in our study. The proportion of allo-HSCT for patients with aggressive ATL was only about 30% in several previous large retrospective analyses [18, 19, 24]. Additionally, the proportion of allo-HSCT patients with aggressive ATL in Osaka, a non-endemic area, was low (approximately 30%) [21].

Baseline ECOG PS may have influenced transplant feasibility. In our study, only one patient with ECOG PS 2–4 at initial treatment underwent allo-HSCT, suggesting that poor ECOG PS at initial treatment limited eligibility. This is consistent with a report by Ito et al. reporting fewer patients with poor ECOG PS in the allo-HSCT group [25]. Although our study included fewer patients with poor ECOG PS, the allo-HSCT implementation rate was lower than that reported by Ito et al. [25]. This suggests that other factors, such as a longer coordination period or early disease progression, may have contributed to the limited transplant feasibility in our study. Moreover, referral bias cannot be ruled out because patients in poor condition may have been less likely to be referred for or selected to undergo allo-HSCT compared with other studies. Although the coordination period in our study was not particularly short, these factors may have contributed to the high allo-HSCT implementation rate.

Ito et al. reported a high implementation rate of 80% for allo-HSCT. The 2 year OS rates from diagnosis were 45.1% for all patients, 47.8% for transplanted patients, and 34.8% for non-transplanted patients, suggesting a trend toward the improved survival of patients who received allo-HSCT after responding to initial chemotherapy [25]. Our study yielded a prognosis comparable with that of Ito et al. However, the median coordination period in their report was 128 days, which was comparatively shorter than our coordination period. In our study, most patients received allo-HSCT from unrelated donors, and the median interval from initial treatment to allo-HSCT through the JMDP was 185 days. Thus, the coordination period of our study was longer than that of Ito et al., and 15 patients who eventually developed PD were unable to undergo allo-HSCT. One of the reasons for the shorter coordination period in their study was the increased use of alternative donors such as cord blood and stem cells from human leukocyte antigen-haploidentical-related donors. The utility of allo-HSCT using alternative donors has also been reported [26–29]. ATL is often resistant to conventional chemotherapeutic agents, and shortening the coordination period is critical to improve the implementation rate of allo-HSCT. In our study, among the 15 patients who could not proceed to allo-HSCT because of PD, two were refractory to initial treatment, two experienced relapse after an initial response, and the remaining 11 developed recurrent disease during the coordination period. These findings suggest that not all PD cases resulted from primary refractory disease, and that a shorter coordination period might have enabled some of these patients to undergo allo-HSCT.

Second, with a median follow-up of 124.4 months for survivors compared with 32.9 months in a previous study [21], we had one of the longest observation periods among ATL studies of non-endemic areas. This extended follow-up period strengthens the reliability of our survival analysis. In our study, the 3 year OS rate of the allo-HSCT group was 45.7% and the 3 year OS rate after allo-HSCT in the CR/PR group was 51.4%. In contrast, Fuji et al. reported a 2 year OS rate after allo-HSCT of 21.4% in Osaka [21]. Although we cannot compare our study with that report from the same non-endemic area, the clinical outcomes of our patients who underwent allo-HSCT were better than those in the previous report [21]. However, the 2 year cumulative incidence of relapse/progression was similar between the two studies (41.3% vs. 53.6%) [21], and the high relapse/progression rate after allo-HSCT in our study is an important issue. Patients with SD/PD before allo-HSCT had a significantly higher incidence of relapse or progression than patients with CR/PR [30]. In our study, the incidence of relapse/progression after allo-HSCT tended to be higher in the SD/PD group than in the CR/PR group. Thus, disease status at allo-HSCT is a critical factor for relapse/progression after allo-HSCT. As cell adhesion molecule 1 (CADM1) is expressed ubiquitously in HTLV-1-infected cells and CD7 is downregulated based on the oncogenic stage of HTLV-1-infected cells, flow cytometry of CADM1 and CD7 expression in CD4-positive cells is used to detect HTLV-1-infected cells [31–33], termed the HTLV-1 analysis system (HAS-flow). Our previous study suggested the predictive significance of HAS-flow as a quantifiable and measurable residual disease [34]. Therefore, predicting high-risk groups for relapse/progression and therapeutic intervention might be possible by performing HAS-flow before or after allo-HSCT. Actually, patients with ATL cell fractions reduced below 40% by HAS-flow prior to allo-HSCT have been shown to have a favorable prognosis [35].

The 2 year cumulative incidence of NRM in our study was lower than in a previous study (17.6% vs. 42.9%) [21]. Although that study included 39.3% of patients who received Moga treatment before allo-HSCT, and the median interval from the last Moga treatment to allo-HSCT was 44 days [21], we included 21.7% of patients who received Moga treatment before allo-HSCT, with a median interval of 69.5 days. Pre-transplantation Moga treatment was reported to be significantly associated with an increased risk of NRM, and pre-transplantation Moga treatment with an interval of < 50 days to allo-HSCT was associated with an increased risk of NRM [36]. Therefore, the smaller number of patients receiving Moga treatment before allo-HSCT and the longer interval from the last Moga administration to allo-HSCT in our study compared with the previous study may have contributed to the reduction in NRM.

In the later period (2012–2017), approximately one-third of patients received Moga before allo-HSCT. Although not statistically significant, this cohort showed a trend toward worse OS, which may be related to the previously reported increased risk of NRM associated with pre-transplantation Moga [36].

Third, the prognosis in the non-allo-HSCT group was poor. Twenty-five patients did not undergo allo-HSCT. The main reason for this was that the disease status of ATL was PD. Recently, novel agents such as brentuximab vedotin, tucidinostat, and valemetostat have shown efficacy in patients with relapsed or refractory ATL [37–39]. Although these treatments were not evaluated in our study, further investigation into their potential role in improving outcomes or facilitating allo-HSCT in eligible patients may be warranted.

Our study has some limitations. First, it was a retrospective study. Therefore, confounding variables could not be excluded, and some clinical data such as the time from initial treatment to PD were not consistently available. Second, most patients were classified as intermediate-risk according to the modified ATL-PI. Therefore, the prognosis might be better than previously reported. Third, some patients with SD/PD at allo-HSCT underwent allo-HSCT at the discretion of the attending physician. However, there are no clear criteria for determining whether allo-HSCT should be performed.

In conclusion, we demonstrated a high implementation rate of allo-HSCT and good treatment outcomes in patients with ATL aged 70 years or younger treated at our group of institutions in a non-endemic metropolitan city. As the incidence of HTLV-1 infection and ATL is increasing in non-endemic metropolitan cities, core hospitals such as our institutions for ATL treatment in non-endemic metropolitan cities are becoming increasingly important.

## Supplementary Information

Below is the link to the electronic supplementary material.

Supplementary file1 Supplemental Fig. 1 Survival of patients with aggressive ATL after allo-HSCT according to transplant period. ATL adult T-cell leukemia-lymphoma (TIF 1915 KB)

## Data Availability

Original data are available on a collaborative basis upon request.
