# Supplementary figures and images for: Outcomes of patients aged 70 years or younger with aggressive ATL at core hospitals for ATL treatment in Tokyo
Source: Int J Hematol. 2025 Sep 2;123(1):52–62. doi: 10.1007/s12185-025-04057-2 (PMC12812779; doi:10.1007/s12185-025-04057-2)

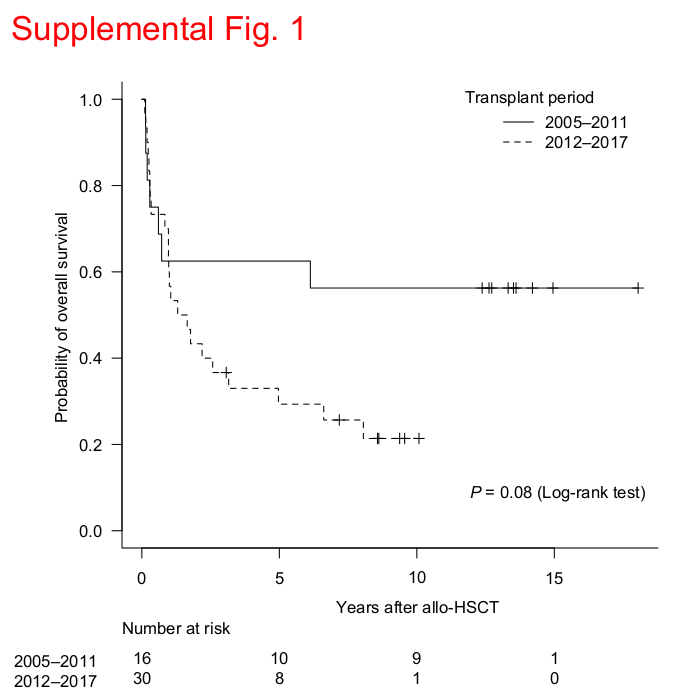

Supplement: Supplementary file 1 — Supplementary file1 Supplemental Fig. 1 Survival of patients with aggressive ATL after allo-HSCT according to transplant period. ATL adult T-cell leukemia-lymphoma (TIF 1915 KB) [file 12185_2025_4057_MOESM1_ESM.tif]
